# Chemokine-Derived Peptides: Novel Antimicrobial and Antineoplasic Agents

**DOI:** 10.3390/ijms160612958

**Published:** 2015-06-08

**Authors:** Julio Valdivia-Silva, Jaciel Medina-Tamayo, Eduardo A. Garcia-Zepeda

**Affiliations:** 1 Chemokine Biology Research Laboratory, Programa Institucional de Investigación en Cancer de Mama, Mexico DF 04510, Mexico; E-Mails: julvalsil@gmail.com (J.V.-S.); jacielmt@gmail.com (J.M.-T.); 2 Departamento de Inmunología, Instituto de Investigaciones Biomédicas, Universidad Nacional Autónoma de México, Mexico DF 04510, Mexico

**Keywords:** chemokine, chemokine receptors, cytokines, inflammation, cancer, microbial infections, peptides

## Abstract

Chemokines are a burgeoning family of chemotactic cytokines displaying a broad array of functions such as regulation of homeostatic leukocyte traffic and development, as well as activating the innate immune system. Their role in controlling early and late inflammatory stages is now well recognized. An improper balance either in chemokine synthesis or chemokine receptor expression contributes to various pathological disorders making chemokines and their receptors a useful therapeutic target. Research in this area is progressing rapidly, and development of novel agents based on chemokine/chemokine receptors antagonist functions are emerging as attractive alternative drugs. Some of these novel agents include generation of chemokine-derived peptides (CDP) with potential agonist and antagonist effects on inflammation, cancer and against bacterial infections. CDP have been generated mainly from N- and C-terminus chemokine sequences with subsequent modifications such as truncations or elongations. In this review, we present a glimpse of the different pharmacological actions reported for CDP and our current understanding regarding the potential use of CDP alone or as part of the novel therapies proposed in the treatment of microbial infections and cancer.

## 1. Introduction

Chemokines are members of a superfamily of small proteins of pro-inflammatory mediators and potent leukocyte chemoattractants. They have been implicated in different activities, including regulation of inflammation, haemostasis, angiogenesis, and cell proliferation [1,2]. There are approximately 50 chemokines grouped into four classes based on the characteristics of the first two of the four conserved cysteine residues: CC, CXC, CX3C, and XC. These chemokines induce signalling by binding to either typical seven-transmembrane G protein-coupled receptors or atypical G-protein independent receptors acting in an arrestin-dependent manner [3,4]. The general chemokine structure consists of an elongated N-terminal segment, three antiparallel β-strands, and a C-terminal α-helix. Chemokines have a highly constrained conformation that is stabilized by two disulphide bonds. Correct chemokine folding is essential for specific interactions with their receptors [5,6]. In addition, CXC chemokines have been further subdivided in ELR^+^ and ELR^−^ chemokines, based on the presence or absence of the tripeptide glutamic acid-leucine-arginine (the “ELR” motif) preceding the CXC domain. The ELR^+^ CXC chemokines, such as interleukin-8 (CXCL8/IL-8), are angiogenic, whereas most ELR^−^ CXC chemokines, like Platelet Factor-4 (CXCL4/PF-4) inhibit angiogenesis [7,8,9]. This “ELR” motif appears to be important in the regulation of ligand/receptor interactions on neutrophils [10]. An exception to the relation between the “ELR” motif and angiogenesis is CXCL12/SDF-1, an angiogenic ELR^−^ CXC chemokine [11]. As chemokines play key roles in regulating pathological inflammation and tumorigenesis, chemokine and chemokine receptor antagonists have become valuable therapeutic agents [12]. A greater understanding of the multiple functions of several chemokines may provide insight into the mechanisms used by these molecules to promote disease pathogenesis, which will aid in the development of such therapeutic agents. In addition, an increased knowledge of the mechanisms involved in chemokine-chemokine receptor activation may also be useful in designing novel receptor antagonists.

## 2. Chemokines and Microbial Infections

### 2.1. Antibacterial Activities

More than twenty years ago, two separate studies reported the ability of antimicrobial proteins derived from human neutrophils to induce chemotaxis in monocytes. The first study reported a monocyte-chemotactic activity predominantly in the defensin-like containing fraction of the neutrophil granules [13]; the other study showed a monocyte specific chemoattractant activity mediated by the cationic antimicrobial peptide-37 (CAP-37 or azurocidin). CAP-37 is located together with ten other proteins in the azurophil granules of the neutrophils widely implicated in the killing of microorganisms [14]. These studies were corroborated by Chertov *et al.*, (1996) [15] who reported that defensins and CAP-37 were able to induce chemotaxis not only in monocytes but also in T lymphocyte cells after neutrophil stimulation [15]. These studies not only uncovered a new role of antimicrobial neutrophils-derived proteins as chemotactic molecules, but also allowed the proposition of chemokines as antimicrobial agents. Indeed, this hypothesis was reinforced by subsequent studies where different chemokines apparently showed high antimicrobial activity. For example, Yang *et al.*, (1999) [16] demonstrated that the human β-defensin was selectively chemotactic for cells stably transfected to express the human CCR6, whose effect was blocked by pertussis toxin and by antibodies anti-CCR6, corroborating that β-defensin is an agonist of the chemokine receptor CCR6 [16]. Interestingly, although β-defensins have no sequence homology with chemokines, the native ligand of CCR6 known as CCL20/MIP-3α possesses similar structural features to human β-defensin-2 [17], including the abundance of cationic residues and the presence of disulphide bonds associated with their common biological activities as discussed below [18]; this study also demonstrated that CCL20/MIP-3α was even more potent than human β-defensin-1 and -2 against *Escherichia coli* and *Staphylococcus aureus*.

Another study showed that two peptides derived from human blood α-granules-platelets, which exhibited antimicrobial activity against *Bacillus subtilis*, *E. coli*, *S. aureus*, *Lactococcus lactis* and *Cryptococcus neoformans*, were the result of truncations in the C-terminal region of the chemokine CXCL7/NAP-2 [19]. Furthermore, three closely related chemokines CXCL9/MIG, CXCL10/IP-10 and CXCL11/ITAC, all members of the IFN-γ-inducible tripeptide motif Glu-Leu-Arg (ELR)^−^ CXC group of chemokines, also display antimicrobial activities against *E. coli* and *Listeria monocytogenes.* Similar to human defensins, this activity was inhibited by high concentrations of NaCl corroborating the intrinsic relationship between these molecules [20]. Based on the study of defensins and other antimicrobial peptides, it has been proposed that cationic amino acids in these proteins could play a crucial role in their antimicrobial activity. From this analysis, it was demonstrated that the antimicrobial effects of CCL28/MEC against *Pseudomonas aeruginosa*, *Klebsiella pneumonia* and *Candida albicans* [21] were dependent on the C-terminal region with highly charged amino acids (RKDRK). Concomitantly, the charge reversal and deletion mutations in this region supported this hypothesis, although the C-terminal region was shown to be essential, but not sufficient for full antimicrobial activity of CCL28/MEC [22].

A more comprehensive report [23] analysed the antimicrobial activities of 30 chemokines at a single dose (10 μg/mL) against *E. coli*, *S. aureus*, and *C. albicans*. From all the chemokines tested, only CXCL2/Gro-β, CXCL10/IP-10, CXCL11/I-TAC, CXCL12/SDF-1α, CCL11/eotaxin-1, and CCL13/MCP-4 demonstrated anti-*C. albicans* activity. CXCL1/Gro-α, CXCL2/Gro-β, CXCL3/Gro-γ, CXCL12/SDF-1, CXCL13/BCA-1, CCL1/I-309, CCL13/MCP-4, CCL19/MIP-3β, CCL20/MIP-3α, and XCL1/lymphotactin, were more potent against *E. coli* compared to *S. aureus*. In addition, the antimicrobial activities of two chemokines CCL19/MIP-3β and CCL21/SLC, showed significant differences although both are similar in size, charge and share the same chemokine receptor, CCR7. Thus, CCL19/MIP-3β was active against *E. coli* with no detectable anti-*S. aureus* activity and CCL21/SLC, albeit less potent against *E. coli* than CCL19/MIP-3β, demonstrated potent anti-*S. aureus* activity. However, some discrepancies in regard to their antimicrobial activity have been reported in different studies [20], and the reason for this discrepancy is most likely a result of the use of different antimicrobial assays or different strains of bacteria [23]. Indeed, most of the antimicrobial chemokines are positively charged at neutral pH, exhibiting a pI higher than 8.0 [23]; this again indicates again that cationicity is a valuable feature for antimicrobial chemokines, but it is not sufficient for distinguishing antimicrobial from non-antimicrobial chemokines. In addition to cationicity, other structural characteristics may endow a given chemokine with antimicrobial activity. IFN-γ-inducible antimicrobial chemokines (CXCL9/MIG, CXCL10/IP-10, and CXCL11/I-TAC) contain a C-terminal segment uniquely rich in positively charged amino acids as described before [20]. It was proposed that the heavily cationic tail of IFN-γ-inducible CXC chemokines could be responsible for their antimicrobial activity via interactions with the anionic moieties on the surface of bacteria disrupting their membrane. It is proposed that there is a fundamental structural principle underlying all antimicrobial peptides which is the ability of the molecule to adopt a shape in which clusters of hydrophobic and cationic amino acids are spatially organized in certain sectors of the molecule, an *amphipathic* design [24]. How antimicrobial chemokines are able to discriminate between membranes of bacteria with those of the host cells (epithelial tissue cells) is not completely understood. Selectivity for distinct lipid compositions may be a possibility [25], and membrane polarity or/and asymmetry may also play a role. In eukaryotic cell membranes, negatively charged phospholipids are sequestered in the inner leaflet of the lipid bilayer, and the outer leaflet is composed mostly of uncharged lipids. Therefore, the lipid content of the outer membranes of eukaryotic cells is devoid of electrostatic charge [26]. In contrast, both leaflets of bacterial cell membranes are enriched with acidic phospholipids such as phosphatidyl-glycerol and cardiolipin, making these membranes negatively charged.

Over the years, reports have broadened the antimicrobial spectrum of chemokines previously reported. A recent review summarized the information and showed that of a total of 45 human chemokines, 23 (10 CXC and 13 CC chemokines) were reported to exhibit antimicrobial activity [27]. Although several reports regarding the antimicrobial activities of chemokines remain controversial due to differences in the selected experimental procedures (bacterial species, culture conditions, killing and radial diffusion assays, *etc.*), the evidence is still positive in favour of this novel chemokine’s activity. For example, the chemokine CXCL6/GCP-2 did not support enough evidence as microbicidal in earlier studies [23] but subsequently was demonstrated to be a potent antimicrobial peptide against several Gram-negative and Gram-positive bacteria [28,29]. Recently, the chemokine CXCL14/BRAK which is constitutively expressed in many epithelial tissues, including skin and gastrointestinal tract [30], taste buds of human and mouse tongues [31], and murine lungs, ovary, brain, kidney, and trachea [32], showed significant *in vitro* antimicrobial activities against Gram-positive as well as Gram-negative bacteria, including skin commensals as well as frequent pathogens and *C. albicans* [30]. Since this chemokine seems to have a homeostatic role due its wide expression in different tissues and its down-modulation under inflammatory conditions [33], CXCL14/BRAK could be a key regulator in antimicrobial immunity in early phases to infection. CXCL14/BRAK shares several structural features such as a high density of positive charges at physiological pH as well as a core-structure consisting of three anti-parallel β-strands reminiscent of the β-defensin fold and a C-terminal α-helix that is typical for the cathelicidin LL-37, a potent antimicrobial peptide in humans [34]. Similarly, chemokine CXCL17/DMC, shown as highly expressed in 105 human tissues and cells, was suggested to be a homeostatic, mucosa-associated chemokine. It has antimicrobial activity against *E. coli*, *S. aureus*, *S. enterica serovar typhimurium 14028s*, *L. casei*, *P. aeruginosa*, and *C. albicans* [35]. Because chemokines induce the recruitment of different immune cells whose actions against microorganisms might overwhelm the initial antimicrobial activity *in vivo*, most studies exploring this property has been limited to *in vitro* analysis. However, two interesting studies *in vivo* showed that the germination of *B. anthracis* spores could be inhibited by CXCL10/IP-10 [36,37]. A correlation was established between higher levels of CXCL9/MIG, CXCL10/IP-10, and CXCL11/I-TAC in the lungs of C57BL/6 mice and the resistance of these mice to respiratory *B. anthracis* infection. Furthermore, C57BL/6 mice pre-treated with CXCL9-, CXCL10-, or CXCL11-neutralizing antibodies were more susceptible to *B. anthracis* infection independently of their chemokine receptor CXCR3. As a better understanding of the involved molecular mechanisms of this chemokine-mediated antimicrobial activity, it was found that disruption of the gene *ftsX*, which encodes the transmembrane domain of a putative ATP-binding cassette transporter, affords resistance to CXCL10-mediated antimicrobial effects against this bacterium. In the absence of *ftsX*, CXCL10/IP-10 was unable to localize to its presumed site of action at the bacterial cell membrane, suggesting that chemokines interact with specific, identifiable bacterial components to mediate direct microbial killing [38].

### 2.2. Anti-Viral and Anti-Parasite Activities

It has been recently reported that chemokines are not only anti-bacterial and anti-fungal, but are also effective against other types of microorganism such as viruses and parasites. For example, CCR5 and CXCR4 play an essential role as the main co-receptors for human immunodeficiency virus-1 (HIV-1) entry into the target cell, and their ligands, the human CC chemokines CCL3/MIP-1α, CCL5/RANTES, CCL8/MCP-2 and CXCL12/SDF-1, respectively, inhibit HIV-1 entry [39,40,41,42]. Indeed, vMIP-II (viral macrophage inflammatory protein-II), a chemokine encoded by human herpes virus 8 (HHV-8) [43] displays diverse interactions with both CC and CXC chemokine receptors and inhibits HIV-1 entry mediated through CCR3, CCR5, and CXCR4 [44]. This broad spectrum receptor binding property of vMIP-II is unique among all known viral chemokines and provides useful templates to study chemokine ligand-receptor interaction and/or design novel small-molecule anti-HIV agents. So, synthetic peptides derived from the N-terminus of vMIP-II were used to probe the mechanism of its biological functions [45,46]. One of these peptides is DV1, whose sequence corresponds to N-terminal amino acid residues 1–21 of vMIP-II (LGASWHRPDKCCLG-YQKRPLP). This peptide displayed antagonistic activity against CXCR4, but not CCR5, and selectively inhibited CXCR4-mediated T- and dual-tropic HIV entry [47]. A variant of CCL14/HCC-1 lacking the first eight amino acids, HCC-1_9–74_, has been isolated from human hemofiltrate by Detheux *et al.*, (2000) [48]. This variant is a potent agonist of chemokine receptors CCR1, CCR3, and CCR5, and inhibits infection by CCR5-tropic human immunodeficiency virus type 1 isolates [48].

In addition, two comprehensive studies have shown anti-parasite activity in *Plasmodium falciparum* and *Leishmania mexicana* respectively. The chemokine CXCL4/hPF4, a human defence peptide derived from platelets, was able to destroy malaria parasites inside erythrocytes by selectively lysing the parasite digestive vacuole [49]. Interestingly, CXCL4/PF4 rapidly accumulated only within infected erythrocytes and was required for parasite killing in infected erythrocyte-platelet co-cultures. Additionally, small peptides derived from this chemokine also reduced parasitemia in a murine malaria model. In the second report, Söbirk *et al.*, (2013) [50], analysed the parasiticidal properties of ten human chemokines (CXCL2/Gro-β, CXCL6/GCP-2, CXCL8/IL-8, CXCL9/MIG, CXCL10/IP-10, CCL2/MCP-1, CCL3/MIP-1α, CCL20/MIP-3α, CCL27/CTACK, and CCL28/MEC) against the promastigote form of the protozoan parasite *Leishmania mexicana*. The results indicated that CXCL6/GCP-2, CXCL9/MIG, and CCL28/MEC were the most effective, causing over 80% death, whereas CXCL8/IL-8, CCL2/MCP-1, CCL3/MIP-1α and CCL27/CTACK were the least effective [50].

## 3. Chemokine-Derived Peptides as Antimicrobials

Chemokine-mediated bacterial killing is completely unrelated to chemokine receptor specificity and appears to involve protein structures that are different from chemokine receptor binding motifs. In addition, other molecules could overshadow the antimicrobial effect of chemokines during inflammation and infection due to the fast recruitment of immune cells. Regarding this, chemokine-derived peptides with specific actions would be an excellent tool to evaluate the antimicrobial activity without other concomitant effects. Essentially, although not all chemokines possess antimicrobial activity, some peptides derived from chemokines are able to kill bacteria. Table 1 summarizes some of the current knowledge of the antimicrobial activities of the chemokine-derived peptides.

**Table 1:** Antimicrobial activity of Chemokine-derived peptides (CDP).

CDP	Chemokine	Region	Biological Target	References
vMIP-II r1-21	vMIP-II	N-terminal	Inhibition of HIV-1 entry	[45]
MIP-3α_51–70_	CCL20/MIP-3α	C-terminal	*E. coli* (ML35)	[23,51]
MIP-3α_59–70_	CCL20/MIP-3α	C-terminal	*E. coli* (ML35)	[52]
TC-1	CXCL7/NAP-2	C-terminal	*E. coli* (ML35) *B. subtilis* (ATCC6633) *S. aureus* (42D) *C. neoformans* (CI) *C. glabrata* (CI)	[51]
IL-8_81–99_	CXCL8/IL-8	C-terminal	*E. coli* (MG1655) *S. enterica* (MS10) *K. pneumoniae* (CCUG 49243) *H. pylori* (SS1) *S. pyogenes* (CCUG 49246)	[58]
CCL13_57–75_ CDAP-4	CCL13/MCP-4	C-terminal	*E. coli* (ATCC25922) *S. typhimurium* (ATCC 14028) *S. typhi* (ATCC 9993) *K. pneumonia* (PIMM-UNAM28) *P. aeruginosa* (ATCC15692) *P. aeruginosa* (CI)	[59]
RP-1	CXCL4/PF4	C-terminal	*L. major* *L. infantum chagasi* (MHOM/BR/00/1669) *L. braziliensis* (CI)	[60]
PMX207, PMX1207	CXCL4/PF4	C-terminal	*P. falciparum* (3D7) *E. coli* (ATCC 25922) *S. aureus* (ATCC 27660) *E. faecalis* (ATCC 29212) *P. aeruginosa* (ATCC 10145) *K. pneumoniae* (ATCC 13883)	[61]

CI: Clinical Isolate.

Peptides derived from the human chemokines macrophage inflammatory protein-3α (CCL20/MIP-3α), 0interleukin-8 (CXCL8/IL-8), and those generated through N-terminal proteolytic processing from platelet basic protein (PBP) including neutrophil activating protein-2 (CXCL7/NAP-2) and thrombocidin-1 (TC-1) have demonstrated antimicrobial activity [51,52,53]. PBP and its derivatives are part of the platelet microbicidal proteins (PMPs), which also include platelet factor-4 (CXCL4/PF4) [54,55]. The platelet chemokines neutrophil activating peptide-2 (CXCL7/NAP-2) and thrombocidin-1 (TC-1) differ by only two amino acids at their carboxyl-terminal ends (truncation of an alanine and aspartate residues) [56]. Nevertheless, they display a significant difference in their direct antimicrobial activities, with the longer CXCL7/NAP-2 being inactive and TC-1 being active. In activated platelets, CXCL7/NAP-2 and the connective tissue activating peptide III (CTAP-III), another PBP-derived peptide typically less active as neutrophil activator, can become proteolytically truncated at the C-terminus by two amino acids to generate thrombocidin-1 (TC-1) and TC-2, respectively [19]. This minor change has significant functional consequences because the thrombocidins have direct bactericidal activities against *B. subtilis*, *E. coli*, *S. aureus*, and *L. lactis* and fungicidal activity against *Cryptococcus neoformans*, whereas intact CXCL7/NAP-2 and CTAP-III do not. These antimicrobial activities have been shown to have important roles for the body’s defence against infections [57]. Similarly, synthetic peptides representing the carboxyl-terminal part of CXCL8/IL-8 possess antibacterial activity against *E. coli*, and *S. pyogenes*, among others [58], while CXCL8/IL-8 does not. Significantly, the CXCL8/IL-8-derived peptide lacked the pro-inflammatory effects of the full-length protein. Moreover, several studies oriented to elucidate the mechanism of action of the antimicrobial chemokines and to determine the key residues involved, have discovered and developed new antimicrobial peptides. Our group has also reported that CDAP-4, a chemokine-derived peptide from CCL13/MCP-4 chemokine, displayed significant antimicrobial activity against *S. typhimurium*, *S. typhi* and several *P. aeruginosa* clinical isolates [59].

Two synthetic peptides RP-1 and AA-RP-1, based on the conserved α-helical domain of CXCL4/PF4, possess activity against three species of *Leishmania*, both *in vitro* as *in vivo*, reducing the liver and spleen parasite load in a model of visceral disease [60]. RP-1 is an 18-amino-acid peptide (*N*-ALYKKFKKKLLKSLKRLG-*C*) modelled in part upon α-helical C-terminal microbicidal domains of mammalian CXCL4 kinocidins. AA-RP-1 is an anthryl-alanine-substituted (position 2) congener of the parent peptide RP-1 [61]. Similarly, peptides derived from the C-terminal region of CXCL4/hPF4, called PMX207 and PMX1207, were able to suppress parasitemia and increased mouse survival in a murine malaria model [49].

It is noteworthy that CDP can differ in their antimicrobial activity compared to the chemokine from which they were derived. For example, the full-length CXCL6/GCP-2 was compared with deletion variants composed of either the C-terminal 19 amino acids or the NH_2_-terminal 50 amino acids. Both peptides displayed lower antimicrobial activity than the full-length CXCL6/GCP-2. However, the NH_2_-terminal peptide was the more potent of the two variant peptides and was able to induce in a sodium chloride-sensitive fashion membrane damage in a model of carboxyfluorescein-loaded liposomes [62]. Another example is the proteolytic activity of the serine protease CD26/dipeptidyl-peptidase IV (CD26/DPP IV) which is known to cleave dipeptides from the NH_2_ terminus of different peptides as chemokines [63]. This protease removes Gly_1_-Pro_2_ dipeptide from the NH_2_ terminus of macrophage-derived chemokines (CCL22/MDC) and subsequently also the Tyr_3_-Gly_4_ dipeptide, generating MDC_(5–69)_. Compared with intact MDC_(1–69)_, CD26/DPP IV-processed MDC_(5–69)_ had reduced chemotactic activity on lymphocytes and monocyte- derived dendritic cells and showed impaired mobilization of intracellular Ca^2+^ through CC chemokine receptor 4 (CCR4); however, MDC_(5–69)_ remained equally chemotactic as intact MDC_(1–69)_ on monocytes and retained its anti-HIV-1 activity. The antimicrobial activity of this fragment have not been evaluated [63].

So far, the structure of the antimicrobial peptides has been related to the three-dimensional amphipathic architecture consisting of a hydrophobic region and a positive patch [18,64]. An amphipathic structure is supposedly required for microbicidal activity, with hydrophobic domains being essential for membrane interactions and cationic domains providing selective interaction with the negatively charged outer surfaces of microorganisms as previously described [23,53]. However, several antimicrobial chemokines as well as disulphide-containing antimicrobial peptides retain antimicrobial activity when linearized [22]. Furthermore, reduction of disulphide bonds and unfolding are required for the full antimicrobial activity of human β-defensin-1 [65]. Therefore, these observations question the necessity of the three-dimensional positive patch for antimicrobial activity and suggest that other structural elements are involved in these activities [51].

In summary, development of antimicrobial peptides derived from chemokines may represent a new pharmacological tool against clinically relevant pathogens that have developed resistance to antimicrobial agents. As chemokines show *in vitro* antimicrobial effectiveness at concentrations ranging from nanomolar to micromolar, it is not clear whether these concentrations are also achievable *in vivo*. By using chemokine derived peptides it is possible to circumvent this problem, since it is possible to achieve similar biological effects by reducing concentrations to nanomolar levels. However, the exact molecular mechanisms of action of these peptides are not fully understood and are under investigation.

## 4. Chemokines, Chemokine-Derived Peptides and Cancer

### 4.1. Paradoxical Roles of Chemokines in Cancer

It is known that prolonged inflammation facilitates carcinogenesis and can trigger tumor progression by providing a microenvironment that is ideal for cancer cell development and growth. In this regard, inflammation has also a major role in regulating chemokine and chemokine receptor expression. Chemokines may play relevant but contrasting roles during the different steps of genesis and progression in cancer. Indeed, this role is rather complex: some chemokines may favour tumor growth and progression, while others may enhance anti-tumor immunity. For example, while some chemokines affect development indirectly by influencing angiogenesis, tumor–leukocyte interactions, as well as directly influencing oncogenesis, survival and growth, invasion and metastasis [11], many others promote the regression or even eradication of a tumor mass by boosting the immune response against the tumor [1]. Although, this double-edged sword is yet poorly understood, recent evidence shows that both the type of cell infiltration and chemokines involved might be key regulators in this process [66]. Chemokines induce migration of leukocyte subpopulations to tumor sites that may promote antitumor activities (such as cytotoxic T cells or natural killer cells), while other chemokines are responsible for large quantities of deleterious tumor-associated macrophages (TAM) at tumour sites [67].

Another relevant function of chemokines is induction of tumor cell invasion and migration, thereby playing fundamental roles in dictating site-directed metastasis formation [68,69]. In addition, chemokine receptor expression in cancer cells has been related to tissue-specific metastasis that might better explain the non-random patterns of organ-tropism by different tumors [70,71]. Our group has provided additional information about cytokine regulation of chemokine receptor expression in cancer cells in the tumor microenvironment [72,73,74]. Briefly, an up or down regulation of the chemokine receptor expression in a particular subpopulation of cancer cells in the tumor due to specific inflammatory stimuli (as pro-inflammatory cytokines) could lead invasion and metastasis to specific organs where their ligands are present [75].

As described, the tumor microenvironment of many types of cancer, including lung, prostate, colon, melanoma and breast cancer, overexpress an extensive network of chemokines and chemokine receptors [76]. Tumor-associated chemokines have at least five described roles in the biology of primary and metastatic disease: (1) control of leukocyte infiltration into the tumor (e.g., CCL2/MCP-1, CCL5/RANTES) [77,78]; (2) manipulation of tumor immune response (e.g., CCL4/MIP-1β); (3) regulation of angiogenesis (CXC chemokines); (4) actions such as autocrine or paracrine growth and survival factors (e.g., CXCL1, 2, 3, 8); and (5) direct the movement of tumor cell themselves (e.g., CXCL12, 19, 21) [79]. A deeper understanding of the complex biology of the tumor microenvironment, and the new roles of the chemokines and their receptors will help to elucidate attractive new therapeutic intervention in cancer patients, including the use of chemokine-derived peptides.

### 4.2. Inflammation and Cancer

Inflammatory cell infiltration is a common feature of cancer. As pointed out above, cells of both innate and adaptive immunity are actively recruited to the tumor site by chemokines produced by neoplastic and stromal cells [11]. CCL2/MCP-1, CCL5/RANTES and ELR^+^ CXC chemokines (ELR motif: glutamic acid-leucine-arginine) have shown pro-tumor activity by recruiting and inducing myeloid cells to differentiate into tumor-associated macrophages (TAM) that exert pro-growth activity inducing neoangiogenesis and inhibition of development of anti-tumor T-cell responses *in situ*. These chemokines play a role in the recruitment of myeloid derived suppressor cells (MDSC) into tumors, and polymorphonuclear leukocytes (PMN) that can acquire a pro-tumor phenotype in the tumor microenvironment [80]. Even more, CCL17/TARC and CCL22/MDC produced by both tumor and infiltrating cells in different types of cancer are able to recruit CCR4^+^ regulatory T and polarized Th_2_ cells that inhibit anti-tumor responses contributing to tumor survival [81,82]. Importantly, some studies have shown contradictory results where CCL2/MCP-1, CCL5/RANTES, CCL17/TARC and CCL22/MDC contribute to the *de novo* formation of tertiary lymphoid structures (TLS) associated with patient long-term survival, and are involved in recruitment of immune cells with antitumor activity [67].

On the contrary, chemokine ligands of CXCR3, CXCR6 and CX3CR1 attract NK cells and T lymphocytes that can elicit anti-tumor responses. Gastric and colorectal carcinoma highly infiltrated by CXCR3 positive lymphocytes and those overexpressing CX3CL1 have a better prognosis than those with normal levels [83]. CXCL16/SR-PSOX has been described as a positive prognostic marker in renal and in colorectal carcinoma, where tumors with high CXCL16/SR-PSOX expression had an increased number of CD4 and CD8 T cells and a better prognosis than the weak CXCL16/SR-PSOX expression group [84].

### 4.3. Angiogenesis Regulation

Angiogenesis is a biological process through which blood vessels are generated. Solid tumor growth requires the presence of neovascularization to guarantee an adequate supply of oxygen and nutrients, growth, and progression. It is well established that ELR^+^ CXC chemokines, such as CXCL1/GRO-α, CXCL2/GRO-β, CXCL3/GRO-γ, CXCL5/ENA-78, CXCL6/GSP-2, CXCL7/NAP-2, and CXCL8/IL-8, have shown to be potent angiogenic factors directly acting on CXCR1 and CXCR2 expressed on endothelial cells stimulating their proliferation, chemotaxis, and inhibiting apoptosis. Via CXCR2, ELR^+^ CXC chemokines induce up-regulation of metalloproteases such as MMP-2 and MMP-9, which are involved in extracellular matrix degradation and release of other angiogenic factors such as VEGF and FGF2 [85]. In contrast, the ELR^−^ CXCL12/SDF-1 chemokine has shown to promote cell migration and proliferation acting synergistically with VEGF via CXCR4 and/or CXCR7 receptors expressed in endothelial cells within the tumor microenvironment [86,87]. Indirectly, these chemokines can also stimulate angiogenesis and this function might be influenced by the type of inflammatory cells recruited into the tumor, as described above. On the other side, ELR^−^ CXC chemokines, such as CXCL4/PF4, CXCL9/MIG, CXCL10/IP-10, CXCL11/I-TAC, CXCL13/BCA-1 and CXCL14/BRAK have demonstrated angiostatic activity in different experimental models and clinical tumors [11,88]. Targeting specific chemokines derived from tumours that may affect tumour angiogenesis is a promising area for the future.

### 4.4. Tumor Progression

Different studies have shown that tumor cells can modulate chemokine systems to promote tumor progression, including invasion and metastasis. For example, the oncogene signalling of the epidermal growth factor receptor (EGFR) via RAS pathway leads to the up-regulation of a distinct set of chemokines, including CXC-chemokine ligand 1 (CXCL1/Gro-α), CXCL8/IL-8 and/or CC-chemokine ligand 20 (CCL20/MIP-3α). These chemokines can stimulate tumor cell proliferation in an autocrine manner or induce angiogenesis [89]. In addition, oncogene signalling might down-modulate the expression of homeostatic chemokines such as CCL27/CTACK and CXCL14/BRAK in order to prevent the recruitment of effector cytotoxic lymphocytes [90,91]. In the same manner, oxysterols produced by tumor cells inhibit the CCR7 expression by mature dendritic cells (DCs) in the surrounding tissue, which prevents their migrating to draining lymph nodes and consequently the antitumor immune responses [92]. As a result, the accumulation of this suppressive infiltrate defeats antitumor immunity and sustains the tolerogenic microenvironment that fosters tumor progression.

On the other hand, tumor cells up-regulate the expression of several chemokine receptors with the aim of colonizing distant sites and setting up a new metastatic homing [93]. Indeed, CXCR4 is overexpressed in more than 20 different tumor histotypes and its involvement in metastasis was broadly demonstrated [70,94]. CXCR1-CXCR5 are associated to aggressive disease and poor prognosis in different tumors such as glioblastoma [95], colorectal carcinoma [96], breast cancer [70], B-cell chronic leukaemia [97] and specific metastasis to liver [98], lymph nodes [99], lung [100], and bone [101]. Moreover, CCR6 drives liver and adrenal metastasis in colorectal carcinoma and lung cancer respectively [102,103], CCR4 and CCR10 are involved in skin metastasis by melanoma and cutaneous lymphoma [104], and CCR9 is associated with intestinal melanoma metastasis [105,106]. It appears that CCR7 and CXCR4 receptors are prominently expressed in cancer and more specifically in metastasis [68,107].

Additionally, two atypical chemokine receptors which are characterized by their inability to transduce conventional signalling, but are able to regulate immune responses by acting as chemokine decoy/scavengers or transporters, the Duffy antigen receptor for chemokines (DARC) and the chemokine scavenger receptor D6, have shown anti-tumoral activity associated with tumor necrosis, decreasing of CC chemokines and MMP9, promoting angiostasis by scavenging of CXC chemokines, and addressing senescence signalling to tumor principally through KAII/CD82 [108,109]. These observations suggested that these receptors expressed by tumor cells or by lymphatic vessels of tumor stroma act as a tumor suppressor gene by negative regulation of pro-tumor chemokine availability.

### 4.5. Chemokine-Derived Peptides with Anti-Tumour Activity

**Figure 1:**
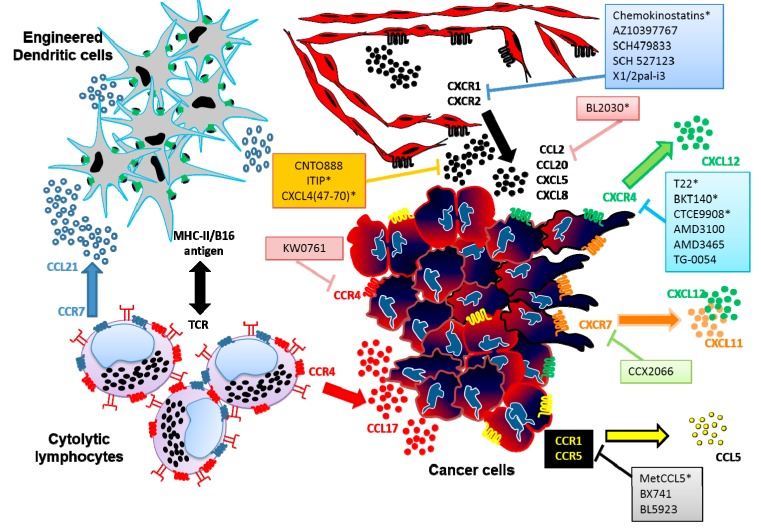
Chemokine-derived peptides (CDP) as novel tools in anticancer therapy. The figure shows three strategies against tumour biology: (1) induction of migration of antitumoral T cells; (2) inhibition of angiogenesis; and (3) reducing tumour growth, invasion and organ-specific metastasis. TCR = T-cell receptor, * chemokine-derived peptides, CTL: Cytotoxic Lymphocytes.

Several chemokine-based strategies, such as antagonist or blocking antibodies and/or small peptides, have been used in different *in vitro* and *in vivo* experiments against cancer so far. Some preclinical trials have demonstrated strong evidence in reducing tumour growth, deleterious cellular infiltrate, vascularity, and metastasis [93] (Figure 1).

An important fact that has been considered in these strategies is the strict correlation between infiltration of the primary tumours by memory T cells, mainly Th1 and CTLs, and the good prognosis and survival in patients at all disease stages. This relation has allowed different experiments and clinical trials involving adoptive cell transfer of *in vitro* expanded anti-tumour specific lymphocytes. However, different studies have shown that less than one percent of the total transferred T cells migrated to the tumor due to mechanisms that remain poorly understood. In this regard, strategies aimed at boosting the migration of T cells into the tumour using chemokines and their receptors could have high relevance in cancer immunotherapy [110]. For example, B16 melanoma lysate-pulsed dendritic cells (DCs) engineered to produce CCL21 and injected into the tumour showed the formation of tertiary lymphoid structures (TLS) containing T cells with antitumor activity [111]. Alternatively, transducing chemokine receptors specific for tumour-produced chemokines in anti-tumour lymphocytes have improved homing and antitumor response *in vivo*; for example T cells equipped with chimeric receptors such as CCR4, the receptor for CCL17, resulted in a strong response against Hodgkin’s tumour models [112].

Probably the most valuable strategy to limit tumour progression is related to blocking chemokines and their receptors since they are directly involved in the more devastating steps in cancer: angiogenesis, invasion, and organ-specific metastasis [93,113]. Indeed, neutralizing anti-CXCL5 and anti-CXCL8 antibodies reduced tumour growth, vascularity, and metastasis in experimental models of non-small cell lung cancer [114]. Also, anti-CCL20 neutralizing antibodies inhibited the growth of prostate cancer cells that overexpress CXCR4 in a tumour xenograft model [115]. Administration of monoclonal antibodies against CCL2 reduced recruitment of inflammatory monocytes and inhibited metastasis to lung in breast cancer, prostate cancer and it is currently being evaluated in ovarian cancer [116,117]. The neutralizing antibody to CCL2, named as CNTO888, decreased tumour burden and bone resorption in a mouse model of prostate cancer [118] and combined with chemotherapy showed improved survival in pre-clinical studies [119]. Furthermore, the soluble receptor anti-CCL2 named BL-2030, derived from the third extracellular domain of CCR2, inhibited prostate tumour growth in the immune-deficient mice SCID [120].

On the other hand, the convergence of the CXCL12/CXCR4 axis in several studies of multiple tumors suggested the use of CXCR4 inhibitors in the treatment of cancer [121]. Small peptides including those derived from the chemokine CXCL12/SDF-1, have been designed and used primarily against the human immunodeficiency virus (HIV) since CXCR4 was found to function as a co-receptor for the entry of T-tropic strains of HIV in CD4^+^ T lymphocytes [122]. Thus, preclinical data showed that inhibition of CXCR4 by neutralizing antibodies, siRNAs, and antagonists such as AMD3100 (Plerixafor, Mozobil) and MSX-122 (Metastatix, phase I trial) can inhibit tumor growth and reduce metastasis [123,124]. Notably, AMD3100 is used in combination with G-CSF for hematopoietic stem cell mobilization for autologous transplantation purposes since it was approved by the FDA in 2008 [125]. Another CXCR4 antagonist, POL6326 has successfully completed Phase I clinical trials and is currently being investigated as a stand-alone therapy in a Phase II clinical trial for its efficacy in autologous transplantation of hematopoietic stem cells in multiple myeloma patients. Interim results of the Phase II trial revealed that POL6326 is safe and well tolerated by all enrolled patients. Likewise, the CXCR4 antagonist TG-0054 (Burixafor, ChemoCentryx) and the aptamer Nox-A12 (Spiegelmer, Noxxon), an anti-CXCL12/SDF-1, are currently being tested in clinical trials in combination with chemotherapy for multiple myeloma and for chronic lymphatic leukaemia [126].

Chemokine-derived peptides based on the N-terminal region sequences, such as T22, BKT140 (4F-benzoyl-TN14003), and CTCE-9908, prevented primary tumour growth and metastasis of melanoma, osteosarcoma, breast and prostate tumours through the inhibition of VEGF production, as well as inhibited angiogenesis and reduced recruitment of myeloid cells [127,128]. T22 ((Tyr (5,12), Lys7)-polyphemusin II) is an 18-residue peptide amide, which takes an antiparallel beta-sheet structure that is maintained by two disulphide bridges. Structure-activity relationship (SAR) studies on T22 have revealed the contributions of each region of T22 to either activity or cytotoxicity. Also, these studies have provided useful information to develop new CXCR4 antagonists: the number of Arg residues in the N-terminal and C-terminal regions is closely related to a better antagonist activity [129]. BKT140 is a 14-residue bio-stable synthetic peptide, which binds CXCR4 with a greater affinity compared with Plerixafor (4 *vs.* 84 nmol/L). Studies in mice demonstrated the efficient and superior mobilization and transplantation of stem cells collected with a mixture of G-CSF and BKT140, compared with those obtained when using stem cells obtained with each one of these mobilizing agent alone. Additionally, BKT140, inhibited primary tumor growth and metastasis of head and neck cancer, and showed a CXCR4-dependent preferential cytotoxicity towards malignant cells of hematopoietic origin in multiple myeloma [126]. A clinical phase I study is now under way using BKT140 in multiple myeloma [130]. CTCE-9908 is a 17 amino acid synthetic peptide CXCR4 antagonist that has been critical in the infiltration of organ tissues by metastatic cells. This drug has the orphan drug status by FDA since 2005 in the treatment of osteogenic sarcoma.

Recently, the specific CXCR4 inhibitor, AMD3465, showed reduction in breast cancer cell invasiveness *in vitro*, infiltration of myeloid CD11b^+^ cells at metastatic sites *in vivo*, and promoted marked changes in oncogenic signalling proteins such as diminished expression of STAT3, JAK2, GSK3, cMYC, and CXCR4 phosphorylation [131]. CXCR4 inhibition may not be sufficient to block the effects of CXCL12, which may also bind to CXCR7 on cancer or stromal cells. In fact, blockade of CXCR4 only partially inhibited migration of cancer cells to CXCL12 gradients *in vivo* [124] because the relationship between CXCR7 (the secondary receptor of CXCL12) and CXCR3 via their shared ligand CXCL11 has made this interaction more complex. Indeed, individual inhibitors of CXCR4, CXCR7 (CCX2066, ChemoCentryx), and CXCR3 (AMG487) showed partial effectiveness in reducing tumour growth and metastasis [132] and they are still in preclinical studies. In addition, CXCL12-derived peptides, including the N-terminal amino acid sequence KPVSLSYR, were used as carriers for gene delivery to CXCR4 expressing cells demonstrating that this technique may be useful in gene therapy of tumor cells expressing CXCR4 [126,133]. Thus, a better understanding of the interactions between these receptors and their ligands can provide novel insights into how to combine these inhibitors effectively and safely for improved cancer therapies.

Similarly, the CCR1 inhibitor BX-471 and the CCR1 antagonist BL5923 were able to decrease myeloma growth [134] and inhibit colon cancer liver metastasis [135] respectively. In addition, a modified version of CCL5, Met-CCL5 with an antagonistic activity on CCR1 and CCR5, showed antitumor effect reducing infiltrating inflammatory cells in a breast cancer model [136]. The humanized defucosylated antibody to CCR4 (KW-0761, Phase II), was demonstrated to have antitumor activity in cutaneous T lymphoma and T-cell acute lymphoblastic leukaemia since the blockade of this receptor showed increased number of CD56 NK cells in the tumour microenvironment [137,138].

As described above, CXC chemokines containing the ELR motif act as pro-angiogenic agents by regulating both endothelial cell proliferation and migration. Interestingly, a set of new peptides derived from CXC chemokines, thrombospondin-1 domain-containing proteins, and IV collagen exhibited notable anti-proliferative and anti-migratory activity *in vitro* and *in vivo* [139,140,141]. Indeed, six 22–24-aminoacid peptides derived from CXC chemokines (CXCL1, 3, 5, 6, 7, and 8) called “chemokinostatins” share similarities to short peptides derived from platelet factor 4 (CXCL4/PF4), a well-established angiogenesis inhibitor [88,142,143]. These peptides showed lower *in vitro* anti-angiogenic activity in human umbilical vein endothelial (HUVEC) cells compared to that obtained by the short peptide derived from CXCL4/PF4 (NGRKICLDLQAPLYKKIIKKLLES) [140]. CXCL4L1, a CXCL4 variant with three amino acid substitutions at P58L, K66E, and L67H in the C-terminus, has been identified as a potent anti-angiogenic chemokine [144]. Although CXCL4 and CXCL4L1 genes are both localized on chromosome 4, the CXCL4L1 gene arises from recent duplication of the CXCL4 gene [145]. The minor difference in their primary structure creates substantial functional differences, including enhanced anti-angiogenic activity, reduced GAG binding, and an increase in the inhibition of endothelial cell migration [145]. Compared with CXCL4, CXCL4L1 is also more effective in inhibiting FGF-2-induced chemotaxis and angiogenesis [146,147]. The C-terminal region (amino acid residues 47–70) of CXCL4L1 has proved to be essential for significant anti-angiogenic and anti-tumor activities, whereas a comparable CXCL4-derived peptide demonstrated less effect [148].

Similarly, orally active small molecule antagonists for CXCR2 and CXCR1 (SCH-479833 and SCH-527123) inhibited human melanoma growth [149] and colon carcinoma liver metastasis in nude mice [150]. In addition, the pepducin named X1/2pal-i3, a palmitoylated peptide based on the structure of the third intracellular loop of CXCR1 and CXCR2 inhibited ovarian tumour growth and angiogenesis [151]. Pepducins are novel cell-penetrating peptides that act as intracellular modulators of signal transference from receptors to G proteins. Using the CXCR2 antagonist AZ10397767 indicates that it inhibited NF-κB mediated evasion of apoptosis in prostate cancer [152]. Finally, a chemokine chimeric molecule designated as ITIP, which was engineered by substituting the N-terminal and N-loop region of CXCL10 with those of CXCL11, led to stronger synergistic antitumor effects than the chemokines alone or in combination *in vitro* and *in vivo* [153] (Summarised in Table 2).

**Table 2:** Chemokine-derived peptides and related molecules with antitumoral activity.

Chemokine	Receptor Inhibitor	Receptor Antagonist	CDP	Other Strategies	Biological Effects and Clinical Trials
CCL2 CCL5 CCL20	Carlumab (CNTO888) [116,117,118,119] Anti-CCL20 [115]	BL5923 [135] BX-471 [134]	Met-CCL5 [136]	BL2030 (a soluble CCR2 receptor fragment) [120]	Regulate leukocyte infiltration Inhibition of TAM formation Improve TLS formation CNTO888 (Phase II) BL5923 and BX471 (Preclinical) BL2030 (Preclinical data)
CCL4 CCL17 CCL22	Mogamulizumab (KW-0761) [137,138]	-	-	Cytotoxic lymphocytes with CCR4 chimeric receptor	Modulation of tumor immune response Inhibition of migration of CCR4^+^ regulatory T cells Improvement of TLS formation Regulation of leukocyte infiltration KW-0761 (Phase II)
CCL21 CCL19 CXCL13	-	-	-	Enhanced production	Improvement of TLS formation [148]
CXCL1 CXCL2 CXCL3 CXCL5 CXCL6 CXCL7 CXCL8	Anti-CXCL5, Anti-CXCL8 [114]	SCH-479833 Navirixin (SCH-527123) AZ10397767 [150,151,153]	Chemokinostatins Pepducin X1/2pal-i3 [88,139,140,141,142,143,152]	-	Inhibition of tumor growth and survival Anti-angiogenesis SCH527123 (Phase I and Phase II) SCH-479833, AZ10397767, X1/2pal-i3 and chemokinostatins (Preclinical)
CXCL4L1	-	-	CXCL4L1/PF4var_47–70_ [149]	-	Anti-angiogenesis Preclinical studies
CXCL4 CXCL9 CXCL10 CXCL11 CXCL13 CXCL14	AMG487 [132]	-	CXCL4/PF4(_47–70_) [140] Chimeric ITIP [154]	-	Tumor growth inhibition Anti-angiogenesis AMG487, ITIP and PF4_47–70_ (Preclinical)
CXCL12 CCL19 CCL21	AMD3465 CCX2066 [131,132]	AMD3100 (Plerixafor) MSX-122 (Metastatix) POL-6326 TG-0054 (Burixafor) [123,124,125,126]	T22, BKT140 (4F-benzoyl-TN14003), CTCE-9908 [127,128,129,130]	Aptamer NOX-A12 (Spiegelmer) [129] Carrier for gene delivery CXCR4: Nter-KPVSLSYR [133]	Inhibition of tumor growth Decrease metastasis Anti-angiogenesis AMD3100 (FDA approved) MSX-122 (Phase I) POL6326 (Phase II) TG-0054 (Phase II) NOX-A12 (Phase II) BKT140 (Phase I) CTCE-9908 (FDA approved) AMD3465 (Preclinical) CCX2066 (Preclinical)
CXCL16	-	-	*-*	Enhanced production	NK and T lymphocytes recruitment, Antitumoral activities

CDP: Chemokine-derived peptides.

## 5. Conclusions

The discovery that members of the chemokine family have antimicrobial peptide activity and/or antitumor effects, in addition to their prototype chemoattractant functions, suggests different roles in the control of antimicrobial and anticancer immunity and allows the subsequent hypotheses to use and test fragments of different chemokines with higher safety than the native molecules which have systemic effects. Importantly, the role of chemokines in immunity during infection and cancer is complex because these molecules may have paradoxical roles especially in tumour biology where they have both pro-tumoral and antitumoral effects depending on multiple factors regarding tumour microenvironment. In addition, chemokines have long been associated with leukocyte recruitment in cancer and progression, and interesting studies highlight the diversity of cancer related inflammation in different tissues and tumours. Thus, in this review we tried to encompass the current knowledge in the use of chemokines and chemokine-derived peptides in infection and cancer in order to gain a global understanding of the complex interaction between chemokines and these pathological processes, and therefore obtain a better therapeutic exploitation based on the detailed components of the chemokine networking.

## References

[1] Rossi D., Zlotnik A. (2000). The biology of chemokines and their receptors. Annu. Rev. Immunol..

[2] Griffith J.W., Sokol C.L., Luster A.D. (2014). Chemokines and chemokine receptors: Positioning cells for host defense and immunity. Annu. Rev. Immunol..

[3] Bachelerie F., Ben-Baruch A., Burkhardt A.M., Combadiere C., Farber J.M., Graham G.J., Horuk R., Sparre-Ulrich A.H., Locati M., Luster A.D. (2014). International Union of Basic and Clinical Pharmacology. [corrected]. LXXXIX. Update on the extended family of chemokine receptors and introducing a new nomenclature for atypical chemokine receptors. Pharmacol. Rev..

[4] Zweemer A.J.M., Toraskar J., Heitman L.H., IJzerman A.P. (2014). Bias in chemokine receptor signalling. Trends Immunol..

[5] Rollins B.J. (1997). Chemokines. Blood.

[6] Biragyn A., Surenhu M., Yang D., Ruffini P.A., Haines B.A., Klyushnenkova E., Oppenheim J.J., Kwak L.W. (2001). Mediators of innate immunity that target immature, but not mature, dendritic cells induce antitumor immunity when genetically fused with nonimmunogenic tumor antigens. J. Immunol..

[7] Balkwill F. (2004). Cancer and the chemokine network. Nat. Rev. Cancer.

[8] Orimo A., Gupta P.B., Sgroi D.C., Arenzana-Seisdedos F., Delaunay T., Naeem R., Carey V.J., Richardson A.L., Weinberg R.A. (2005). Stromal fibroblasts present in invasive human breast carcinomas promote tumor growth and angiogenesis through elevated SDF-1/CXCL12 secretion. Cell.

[9] Miao Z., Luker K.E., Summers B.C., Berahovich R., Bhojani M.S., Rehemtulla A., Kleer C.G., Essner J.J., Nasevicius A., Luker G.D. (2007). CXCR7 (RDC1) promotes breast and lung tumor growth *in vivo* and is expressed on tumor-associated vasculature. Proc. Natl. Acad. Sci. USA.

[10] Strieter R.M., Polverini P.J., Kunkel S.L., Arenberg D.A., Burdick M.D., Kasper J., Dzuiba J., van Damme J., Walz A., Marriott D. (1995). The functional role of the ELR motif in CXC chemokine-mediated angiogenesis. J. Biol. Chem..

[11] Strieter R.M., Burdick M.D., Gomperts B.N., Belperio J.A., Keane M.P. (2005). CXC chemokines in angiogenesis. Cytokine Growth Factor Rev..

[12] Schall T.J., Proudfoot A.E. (2011). Overcoming hurdles in developing successful drugs targeting chemokine receptors. Nat. Rev. Immunol..

[13] Territo M.C., Ganz T., Selsted M.E., Lehrer R. (1989). Monocyte-chemotactic activity of defensins from human neutrophils. J. Clin. Investig..

[14] Pereira H.A., Shafer W.M., Pohl J., Martin L.E., Spitznagel J.K. (1990). CAP37, a human neutrophil-derived chemotactic factor with monocyte specific activity. J. Clin. Investig..

[15] Chertov O., Michiel D.F., Xu L., Wang J.M., Tani K., Murphy W.J., Longo D.L., Taub D.D., Oppenheim J.J. (1996). Identification of defensin-1, defensin-2, and CAP37/azurocidin as T-cell chemoattractant proteins released from interleukin-8-stimulated neutrophils. J. Biol. Chem..

[16] Yang D., Chertov O., Bykovskaia S.N., Chen Q., Buffo M.J., Shogan J., Anderson M., Schroder J.M., Wang J.M., Howard O.M. (1999). Beta-defensins: Linking innate and adaptive immunity through dendritic and T cell CCR6. Science.

[17] Perez-Canadillas J.M., Zaballos A., Gutierrez J., Varona R., Roncal F., Albar J.P., Marquez G., Bruix M. (2001). NMR solution structure of murine CCL20/MIP-3alpha, a chemokine that specifically chemoattracts immature dendritic cells and lymphocytes through its highly specific interaction with the beta-chemokine receptor CCR6. J. Biol. Chem..

[18] Hoover D.M., Boulegue C., Yang D., Oppenheim J.J., Tucker K., Lu W., Lubkowski J. (2002). The structure of human macrophage inflammatory protein-3alpha/CCL20. Linking antimicrobial and CC chemokine receptor-6-binding activities with human beta-defensins. J. Biol. Chem..

[19] Krijgsveld J., Zaat S.A., Meeldijk J., van Veelen P.A., Fang G., Poolman B., Brandt E., Ehlert J.E., Kuijpers A.J., Engbers G.H. (2000). Thrombocidins, microbicidal proteins from human blood platelets, are C-terminal deletion products of CXC chemokines. J. Biol. Chem..

[20] Cole A.M., Ganz T., Liese A.M., Burdick M.D., Liu L., Strieter R.M. (2001). Cutting edge: IFN-inducible ELR^−^ CXC chemokines display defensin-like antimicrobial activity. J. Immunol.

[21] Hieshima K., Ohtani H., Shibano M., Izawa D., Nakayama T., Kawasaki Y., Shiba F., Shiota M., Katou F., Saito T. (2003). CCL28 has dual roles in mucosal immunity as a chemokine with broad-spectrum antimicrobial activity. J. Immunol..

[22] Liu B., Wilson E. (2010). The antimicrobial activity of CCL28 is dependent on *C*-terminal positively-charged amino acids. Eur. J. Immunol..

[23] Yang D., Chen Q., Hoover D.M., Staley P., Tucker K.D., Lubkowski J., Oppenheim J.J. (2003). Many chemokines including CCL20/MIP-3alpha display antimicrobial activity. J. Leukoc. Biol..

[24] Zasloff M. (2002). Antimicrobial peptides of multicellular organisms. Nature.

[25] Lai Y., Gallo R.L. (2009). AMPed up immunity: How antimicrobial peptides have multiple roles in immune defense. Trends Immunol..

[26] Yung S.C., Murphy P.M. (2012). Antimicrobial chemokines. Front. Immunol..

[27] Wolf M., Moser B. (2012). Antimicrobial activities of chemokines: Not just a side-effect?. Front. Immunol..

[28] Collin M., Linge H.M., Bjartell A., Giwercman A., Malm J., Egesten A. (2008). Constitutive expression of the antibacterial CXC chemokine GCP-2/CXCL6 by epithelial cells of the male reproductive tract. J. Reprod. Immunol..

[29] Linge H.M., Collin M., Nordenfelt P., Morgelin M., Malmsten M., Egesten A. (2008). The human CXC chemokine granulocyte chemotactic protein 2 (GCP-2)/CXCL6 possesses membrane-disrupting properties and is antibacterial. Antimicrob. Agents Chemother..

[30] Maerki C., Meuter S., Liebi M., Muhlemann K., Frederick M.J., Yawalkar N., Moser B., Wolf M. (2009). Potent and broad-spectrum antimicrobial activity of CXCL14 suggests an immediate role in skin infections. J. Immunol..

[31] Hevezi P., Moyer B.D., Lu M., Gao N., White E., Echeverri F., Kalabat D., Soto H., Laita B., Li C. (2009). Genome-wide analysis of gene expression in primate taste buds reveals links to diverse processes. PLoS ONE.

[32] Meuter S., Moser B. (2008). Constitutive expression of CXCL14 in healthy human and murine epithelial tissues. Cytokine.

[33] Frick I.M., Nordin S.L., Baumgarten M., Morgelin M., Sorensen O.E., Olin A.I., Egesten A. (2011). Constitutive and inflammation-dependent antimicrobial peptides produced by epithelium are differentially processed and inactivated by the commensal Finegoldia magna and the pathogen Streptococcus pyogenes. J. Immunol..

[34] Cederlund A., Gudmundsson G.H., Agerberth B. (2011). Antimicrobial peptides important in innate immunity. FEBS J..

[35] Burkhardt A.M., Tai K.P., Flores-Guiterrez J.P., Vilches-Cisneros N., Kamdar K., Barbosa-Quintana O., Valle-Rios R., Hevezi P.A., Zuniga J., Selman M. (2012). CXCL17 is a mucosal chemokine elevated in idiopathic pulmonary fibrosis that exhibits broad antimicrobial activity. J. Immunol..

[36] Crawford M.A., Zhu Y., Green C.S., Burdick M.D., Sanz P., Alem F., O’Brien A.D., Mehrad B., Strieter R.M., Hughes M.A. (2009). Antimicrobial effects of interferon-inducible CXC chemokines against Bacillus anthracis spores and bacilli. Infect. Immun..

[37] Crawford M.A., Burdick M.D., Glomski I.J., Boyer A.E., Barr J.R., Mehrad B., Strieter R.M., Hughes M.A. (2010). Interferon-inducible CXC chemokines directly contribute to host defense against inhalational anthrax in a murine model of infection. PLoS Pathog..

[38] Crawford M.A., Lowe D.E., Fisher D.J., Stibitz S., Plaut R.D., Beaber J.W., Zemansky J., Mehrad B., Glomski I.J., Strieter R.M. (2011). Identification of the bacterial protein FtsX as a unique target of chemokine-mediated antimicrobial activity against Bacillus anthracis. Proc. Natl. Acad. Sci. USA.

[39] Broder C.C., Collman R.G. (1997). Chemokine receptors and HIV. J. Leukoc. Biol..

[40] Berger E.A., Murphy P.M., Farber J.M. (1999). Chemokine receptors as HIV-1 coreceptors: Roles in viral entry, tropism, and disease. Annu. Rev. Immunol..

[41] Yang O.O., Garcia-Zepeda E.A., Walker B.D., Luster A.D. (2002). Monocyte chemoattractant protein-2 (CC chemokine ligand 8) inhibits replication of human immunodeficiency virus type 1 via CC chemokine receptor 5. J. Infect. Dis..

[42] Choi W.T., An J. (2011). Biology and clinical relevance of chemokines and chemokine receptors CXCR4 and CCR5 in human diseases. Exp. Biol. Med..

[43] Kledal T.N., Rosenkilde M.M., Coulin F., Simmons G., Johnsen A.H., Alouani S., Power C.A., Luttichau H.R., Gerstoft J., Clapham P.R. (1997). A broad-spectrum chemokine antagonist encoded by Kaposi’s sarcoma-associated herpesvirus. Science.

[44] Moore J.P., Trkola A., Dragic T. (1997). Co-receptors for HIV-1 entry. Curr. Opin. Immunol..

[45] Zhou N., Luo Z., Luo J., Hall J.W., Huang Z. (2000). A novel peptide antagonist of CXCR4 derived from the N-terminus of viral chemokine vMIP-II. Biochemistry.

[46] Zhou N., Luo Z., Luo J., Fan X., Cayabyab M., Hiraoka M., Liu D., Han X., Pesavento J., Dong C.Z. (2002). Exploring the stereochemistry of CXCR4-peptide recognition and inhibiting HIV-1 entry with d-peptides derived from chemokines. J. Biol. Chem..

[47] Xu Y., Duggineni S., Espitia S., Richman D.D., An J., Huang Z. (2013). A synthetic bivalent ligand of CXCR4 inhibits HIV infection. Biochem. Biophys. Res. Commun..

[48] Detheux M., Ständker L., Vakili J., Münch J., Forssmann U., Adermann K., Pöhlmann S., Vassart G., Kirchhoff F., Parmentier M. (2000). Natural proteolytic processing of hemofiltrate Cc chemokine 1 generates a potent Cc chemokine receptor (Ccr)1 and Ccr5 agonist with anti-HIV properties. J. Exp. Med..

[49] Love M.S., Millholland M.G., Mishra S., Kulkarni S., Freeman K.B., Pan W., Kavash R.W., Costanzo M.J., Jo H., Daly T.M. (2012). Platelet factor 4 activity against *P. falciparum* and its translation to nonpeptidic mimics as antimalarials. Cell Host Microbe.

[50] Sobirk S.K., Morgelin M., Egesten A., Bates P., Shannon O., Collin M. (2013). Human chemokines as antimicrobial peptides with direct parasiticidal effect on *Leishmania mexicana in vitro*. PLoS ONE.

[51] Kwakman P.H., Krijgsveld J., de Boer L., Nguyen L.T., Boszhard L., Vreede J., Dekker H.L., Speijer D., Drijfhout J.W., te Velde A.A. (2011). Native thrombocidin-1 and unfolded thrombocidin-1 exert antimicrobial activity via distinct structural elements. J. Biol. Chem..

[52] Nguyen L.T., Chan D.I., Boszhard L., Zaat S.A., Vogel H.J. (2010). Structure-function studies of chemokine-derived carboxy-terminal antimicrobial peptides. Biochim. Biophys. Acta.

[53] Nguyen L.T., Kwakman P.H., Chan D.I., Liu Z., de Boer L., Zaat S.A., Vogel H.J. (2011). Exploring platelet chemokine antimicrobial activity: Nuclear magnetic resonance backbone dynamics of NAP-2 and TC-1. Antimicrob. Agents Chemother..

[54] Yeaman M.R., Yount N.Y., Waring A.J., Gank K.D., Kupferwasser D., Wiese R., Bayer A.S., Welch W.H. (2007). Modular determinants of antimicrobial activity in platelet factor-4 family kinocidins. Biochim. Biophys. Acta.

[55] Yount N.Y., Gank K.D., Xiong Y.Q., Bayer A.S., Pender T., Welch W.H., Yeaman M.R. (2004). Platelet microbicidal protein 1: Structural themes of a multifunctional antimicrobial peptide. Antimicrob. Agents Chemother..

[56] Walz A., Baggiolini M. (1990). Generation of the neutrophil-activating peptide NAP-2 from platelet basic protein or connective tissue-activating peptide III through monocyte proteases. J. Exp. Med..

[57] Dankert J., Krijgsveld J., van Der Werff J., Joldersma W., Zaat S.A. (2001). Platelet microbicidal activity is an important defense factor against viridans streptococcal endocarditis. J. Infect. Dis..

[58] Bjorstad A., Fu H., Karlsson A., Dahlgren C., Bylund J. (2005). Interleukin-8-derived peptide has antibacterial activity. Antimicrob. Agents Chemother..

[59] Martinez-Becerra F., Silva D.A., Dominguez-Ramirez L., Mendoza-Hernandez G., Lopez-Vidal Y., Soldevila G., Garcia-Zepeda E.A. (2007). Analysis of the antimicrobial activities of a chemokine-derived peptide (CDAP-4) on *Pseudomonas aeruginosa*. Biochem. Biophys. Res. Commun..

[60] Erfe M.C.B., David C.V., Huang C., Lu V., Maretti-Mira A.C., Haskell J., Bruhn K.W., Yeaman M.R., Craft N. (2012). Efficacy of synthetic peptides RP-1 and AA-RP-1 against *Leishmania* species *in vitro* and *in vivo*. Antimicrob. Agents Chemother..

[61] Bourbigot S., Dodd E., Horwood C., Cumby N., Fardy L., Welch W.H., Ramjan Z., Sharma S., Waring A.J., Yeaman M.R. (2009). Antimicrobial peptide RP-1 structure and interactions with anionic *vs.* zwitterionic micelles. Biopolymers.

[62] Linge H.M., Collin M., Giwercman A., Malm J., Bjartell A., Egesten A. (2008). The antibacterial chemokine MIG/CXCL9 is constitutively expressed in epithelial cells of the male urogenital tract and is present in seminal plasma. J. Interferon Cytokine Res..

[63] Proost P., Struyf S., Schols D., Opdenakker G., Sozzani S., Allavena P., Mantovani A., Augustyns K., Bal G., Haemers A. (1999). Truncation of macrophage-derived chemokine by CD26/ dipeptidyl-peptidase IV beyond Its predicted cleavage site affects chemotactic activity and CC chemokine receptor 4 interaction. J. Biol. Chem..

[64] Yount N.Y., Waring A.J., Gank K.D., Welch W.H., Kupferwasser D., Yeaman M.R. (2007). Structural correlates of antimicrobial efficacy in IL-8 and related human kinocidins. Biochim. Biophys. Acta.

[65] Schroeder B.O., Wu Z., Nuding S., Groscurth S., Marcinowski M., Beisner J., Buchner J., Schaller M., Stange E.F., Wehkamp J. (2011). Reduction of disulphide bonds unmasks potent antimicrobial activity of human beta-defensin 1. Nature.

[66] Leibovich-Rivkin T., Liubomirski Y., Bernstein B., Meshel T., Ben-Baruch A. (2013). Inflammatory factors of the tumor microenvironment induce plasticity in nontransformed breast epithelial cells: EMT, invasion, and collapse of normally organized breast textures. Neoplasia.

[67] Viola A., Sarukhan A., Bronte V., Molon B. (2012). The pros and cons of chemokines in tumor immunology. Trends Immunol..

[68] Zlotnik A. (2006). Involvement of chemokine receptors in organ-specific metastasis. Contrib. Microbiol..

[69] Ben-Baruch A. (2008). Organ selectivity in metastasis: regulation by chemokines and their receptors. Clin. Exp. Metastasis.

[70] Muller A., Homey B., Soto H., Ge N., Catron D., Buchanan M.E., McClanahan T., Murphy E., Yuan W., Wagner S.N. (2001). Involvement of chemokine receptors in breast cancer metastasis. Nature.

[71] Lorusso G., Ruegg C. (2012). New insights into the mechanisms of organ-specific breast cancer metastasis. Semin. Cancer Biol..

[72] Valdivia-Silva J.E., Franco-Barraza J., Silva A.L., Pont G.D., Soldevila G., Meza I., Garcia-Zepeda E.A. (2009). Effect of pro-inflammatory cytokine stimulation on human breast cancer: Implications of chemokine receptor expression in cancer metastasis. Cancer Lett..

[73] Franco-Barraza J., Valdivia-Silva J.E., Zamudio-Meza H., Castillo A., Garcia-Zepeda E.A., Benitez-Bribiesca L., Meza I. (2010). Actin cytoskeleton participation in the onset of IL-1beta induction of an invasive mesenchymal-like phenotype in epithelial MCF-7 cells. Arch. Med. Res..

[74] Valdivia-Silva J., Franco-Barraza J., Cukierman E., García-Zepeda E.A., Gunduz M., Gunduz E. (2011). Novel insights Into the role of inflammation in promoting breast cancer development. Breast Cancer—Focusing Tumor Microenvironment, Stem Cells and Metastasis.

[75] Karnoub A.E., Weinberg R.A. (2006). Chemokine networks and breast cancer metastasis. Breast Dis..

[76] Zlotnik A. (2006). Chemokines and cancer. Int. J. Cancer.

[77] Rahir G., Moser M. (2012). Tumor microenvironment and lymphocyte infiltration. Cancer Immunol. Immunother..

[78] Moser B., Willimann K. (2004). Chemokines: Role in inflammation and immune surveillance. Ann. Rheum. Dis..

[79] Koizumi K., Hojo S., Akashi T., Yasumoto K., Saiki I. (2007). Chemokine receptors in cancer metastasis and cancer cell-derived chemokines in host immune response. Cancer Sci..

[80] Fridlender Z.G., Sun J., Kim S., Kapoor V., Cheng G., Ling L., Worthen G.S., Albelda S.M. (2009). Polarization of tumor-associated neutrophil phenotype by TGF-beta: “N1” *vs.* “N2” TAN. Cancer Cell.

[81] Maruyama T., Kono K., Izawa S., Mizukami Y., Kawaguchi Y., Mimura K., Watanabe M., Fujii H. (2010). CCL17 and CCL22 chemokines within tumor microenvironment are related to infiltration of regulatory T cells in esophageal squamous cell carcinoma. Dis. Esophagus.

[82] Mizukami H., Shirahata A., Goto T., Sakata M., Saito M., Ishibashi K., Kigawa G., Nemoto H., Sanada Y., Hibi K. (2008). PGP9.5 methylation as a marker for metastatic colorectal cancer. Anticancer Res..

[83] Musha H., Ohtani H., Mizoi T., Kinouchi M., Nakayama T., Shiiba K., Miyagawa K., Nagura H., Yoshie O., Sasaki I. (2005). Selective infiltration of CCR5^+^CXCR3^+^ T lymphocytes in human colorectal carcinoma. Int. J. Cancer.

[84] Hojo S., Koizumi K., Tsuneyama K., Arita Y., Cui Z., Shinohara K., Minami T., Hashimoto I., Nakayama T., Sakurai H. (2007). High-level expression of chemokine CXCL16 by tumor cells correlates with a good prognosis and increased tumor-infiltrating lymphocytes in colorectal cancer. Cancer Res..

[85] Li A., Varney M.L., Valasek J., Godfrey M., Dave B.J., Singh R.K. (2005). Autocrine role of interleukin-8 in induction of endothelial cell proliferation, survival, migration and MMP-2 production and angiogenesis. Angiogenesis.

[86] Kryczek I., Lange A., Mottram P., Alvarez X., Cheng P., Hogan M., Moons L., Wei S., Zou L., Machelon V. (2005). Cxcl12 and vascular endothelial growth factor synergistically induce neoangiogenesis in human ovarian cancers. Cancer Res..

[87] Vandercappellen J., van Damme J., Struyf S. (2008). The role of CXC chemokines and their receptors in cancer. Cancer Lett..

[88] Maurer A.M., Zhou B., Han Z.C. (2006). Roles of platelet factor 4 in hematopoiesis and angiogenesis. Growth Factors.

[89] Wislez M., Fujimoto N., Izzo J.G., Hanna A.E., Cody D.D., Langley R.R., Tang H., Burdick M.D., Sato M., Minna J.D. (2006). High expression of ligands for chemokine receptor CXCR2 in alveolar epithelial neoplasia induced by oncogenic kras. Cancer Res..

[90] Gao J.Q., Tsuda Y., Han M., Xu D.H., Kanagawa N., Hatanaka Y., Tani Y., Mizuguchi H., Tsutsumi Y., Mayumi T. (2009). NK cells are migrated and indispensable in the anti-tumor activity induced by CCL27 gene therapy. Cancer Immunol. Immunother..

[91] Tessema M., Klinge D.M., Yingling C.M., Do K., van Neste L., Belinsky S.A. (2010). Re-expression of CXCL14, a common target for epigenetic silencing in lung cancer, induces tumor necrosis. Oncogene.

[92] Villablanca E.J., Raccosta L., Zhou D., Fontana R., Maggioni D., Negro A., Sanvito F., Ponzoni M., Valentinis B., Bregni M. (2010). Tumor-mediated liver X receptor-alpha activation inhibits CC chemokine receptor-7 expression on dendritic cells and dampens antitumor responses. Nat. Med..

[93] Zlotnik A., Burkhardt A.M., Homey B. (2011). Homeostatic chemokine receptors and organ-specific metastasis. Nat. Rev. Immunol..

[94] Mukherjee D., Zhao J. (2013). The Role of chemokine receptor CXCR4 in breast cancer metastasis. Am. J. Cancer Res..

[95] Ehtesham M., Mapara K.Y., Stevenson C.B., Thompson R.C. (2009). CXCR4 mediates the proliferation of glioblastoma progenitor cells. Cancer Lett..

[96] Verbeke H., Struyf S., Laureys G., van Damme J. (2011). The expression and role of CXC chemokines in colorectal cancer. Cytokine Growth Factor Rev..

[97] Mohle R., Failenschmid C., Bautz F., Kanz L. (1999). Overexpression of the chemokine receptor CXCR4 in B cell chronic lymphocytic leukemia is associated with increased functional response to stromal cell-derived factor-1 (SDF-1). Leukemia.

[98] Yun J.A., Kim H.C., Kim S.H., Cho Y.B., Yun S.H., Lee W.Y., Chun H.K. (2014). Prognostic significance of perineural invasion in stage IIA colon cancer. ANZ J. Surg..

[99] Kawada K., Hosogi H., Sonoshita M., Sakashita H., Manabe T., Shimahara Y., Sakai Y., Takabayashi A., Oshima M., Taketo M.M. (2007). Chemokine receptor CXCR3 promotes colon cancer metastasis to lymph nodes. Oncogene.

[100] Saur D., Seidler B., Schneider G., Algul H., Beck R., Senekowitsch-Schmidtke R., Schwaiger M., Schmid R.M. (2005). CXCR4 expression increases liver and lung metastasis in a mouse model of pancreatic cancer. Gastroenterology.

[101] Wang J., Loberg R., Taichman R.S. (2006). The pivotal role of CXCL12 (SDF-1)/CXCR4 axis in bone metastasis. Cancer Metastasis Rev..

[102] Ghadjar P., Rubie C., Aebersold D.M., Keilholz U. (2009). The chemokine CCL20 and its receptor CCR6 in human malignancy with focus on colorectal cancer. Int. J. Cancer.

[103] Raynaud C.M., Mercier O., Dartevelle P., Commo F., Olaussen K.A., de Montpreville V., Andre F., Sabatier L., Soria J.C. (2010). Expression of chemokine receptor CCR6 as a molecular determinant of adrenal metastatic relapse in patients with primary lung cancer. Clin. Lung Cancer.

[104] Klein A., Sagi-Assif O., Izraely S., Meshel T., Pasmanik-Chor M., Nahmias C., Couraud P.O., Erez N., Hoon D.S., Witz I.P. (2012). The metastatic microenvironment: Brain-derived soluble factors alter the malignant phenotype of cutaneous and brain-metastasizing melanoma cells. Int. J. Cancer.

[105] Amersi F.F., Terando A.M., Goto Y., Scolyer R.A., Thompson J.F., Tran A.N., Faries M.B., Morton D.L., Hoon D.S. (2008). Activation of CCR9/CCL25 in cutaneous melanoma mediates preferential metastasis to the small intestine. Clin. Cancer Res..

[106] Letsch A., Keilholz U., Schadendorf D., Assfalg G., Asemissen A.M., Thiel E., Scheibenbogen C. (2004). Functional CCR9 expression is associated with small intestinal metastasis. J. Investig. Dermatol..

[107] Meijer J., Ogink J., Roos E. (2008). Effect of the chemokine receptor CXCR7 on proliferation of carcinoma cells *in vitro* and *in vivo*. Br. J. Cancer.

[108] Shen H., Schuster R., Stringer K.F., Waltz S.E., Lentsch A.B. (2006). The duffy antigen/receptor for chemokines (DARC) regulates prostate tumor growth. FASEB J..

[109] Bonecchi R., Savino B., Borroni E.M., Mantovani A., Locati M. (2010). Chemokine decoy receptors: Structure-function and biological properties. Curr. Top. Microbiol. Immunol..

[110] Bobisse S., Rondina M., Merlo A., Tisato V., Mandruzzato S., Amendola M., Naldini L., Willemsen R.A., Debets R., Zanovello P. (2009). Reprogramming T lymphocytes for melanoma adoptive immunotherapy by T-cell receptor gene transfer with lentiviral vectors. Cancer Res..

[111] Baratelli F., Takedatsu H., Hazra S., Peebles K., Luo J., Kurimoto P.S., Zeng G., Batra R.K., Sharma S., Dubinett S.M. (2008). Pre-clinical characterization of GMP grade CCL21-gene modified dendritic cells for application in a phase I trial in non-small cell lung cancer. J. Transl. Med..

[112] Di Stasi A., de Angelis B., Rooney C.M., Zhang L., Mahendravada A., Foster A.E., Heslop H.E., Brenner M.K., Dotti G., Savoldo B. (2009). T lymphocytes coexpressing CCR4 and a chimeric antigen receptor targeting CD30 have improved homing and antitumor activity in a Hodgkin tumor model. Blood.

[113] Ali S., Lazennec G. (2007). Chemokines: Novel targets for breast cancer metastasis. Cancer Metastasis Rev..

[114] Arenberg D.A., Keane M.P., DiGiovine B., Kunkel S.L., Morris S.B., Xue Y.Y., Burdick M.D., Glass M.C., Iannettoni M.D., Strieter R.M. (1998). Epithelial-neutrophil activating peptide (ENA-78) is an important angiogenic factor in non-small cell lung cancer. J. Clin. Investig..

[115] Beider K., Abraham M., Begin M., Wald H., Weiss I.D., Wald O., Pikarsky E., Abramovitch R., Zeira E., Galun E. (2009). Interaction between CXCR4 and CCL20 pathways regulates tumor growth. PLoS ONE.

[116] Loberg R.D., Ying C., Craig M., Day L.L., Sargent E., Neeley C., Wojno K., Snyder L.A., Yan L., Pienta K.J. (2007). Targeting CCL2 with systemic delivery of neutralizing antibodies induces prostate cancer tumor regression *in vivo*. Cancer Res..

[117] Garber K. (2009). First results for agents targeting cancer-related inflammation. J. Natl. Cancer Inst..

[118] Sandhu S.K., Papadopoulos K., Fong P.C., Patnaik A., Messiou C., Olmos D., Wang G., Tromp B.J., Puchalski T.A., Balkwill F. (2013). A first-in-human, first-in-class, phase I study of carlumab (CNTO 888), a human monoclonal antibody against CC-chemokine ligand 2 in patients with solid tumors. Cancer Chemother. Pharmacol..

[119] Rozel S., Galban C.J., Nicolay K., Lee K.C., Sud S., Neeley C., Snyder L.A., Chenevert T.L., Rehemtulla A., Ross B.D. (2009). Synergy between anti-CCL2 and docetaxel as determined by DW-MRI in a metastatic bone cancer model. J. Cell. Biochem..

[120] Izhak L., Wildbaum G., Weinberg U., Shaked Y., Alami J., Dumont D., Friedman B., Stein A., Karin N. (2010). Predominant expression of CCL2 at the tumor site of prostate cancer patients directs a selective loss of immunological tolerance to CCL2 that could be amplified in a beneficial manner. J. Immunol..

[121] Domanska U.M., Kruizinga R.C., Nagengast W.B., Timmer-Bosscha H., Huls G., de Vries E.G., Walenkamp A.M. (2013). A review on CXCR4/CXCL12 axis in oncology: No place to hide. Eur. J. Cancer.

[122] Heveker N., Tissot M., Thuret A., Schneider-Mergener J., Alizon M., Roch M., Marullo S. (2001). Pharmacological properties of peptides derived from stromal cell-derived factor 1: Study on human polymorphonuclear cells. Mol. Pharmacol..

[123] Wong D., Korz W. (2008). Translating an Antagonist of Chemokine Receptor CXCR4: From bench to bedside. Clin. Cancer Res..

[124] Duda D.G., Kozin S.V., Kirkpatrick N.D., Xu L., Fukumura D., Jain R.K. (2011). CXCL12 (SDF1alpha)-CXCR4/CXCR7 pathway inhibition: an emerging sensitizer for anticancer therapies?. Clin. Cancer Res..

[125] Broxmeyer H.E., Orschell C.M., Clapp D.W., Hangoc G., Cooper S., Plett P.A., Liles W.C., Li X., Graham-Evans B., Campbell T.B. (2005). Rapid mobilization of murine and human hematopoietic stem and progenitor cells with AMD3100, a CXCR4 antagonist. J. Exp. Med..

[126] De Nigris F., Schiano C., Infante T., Napoli C. (2012). CXCR4 inhibitors: tumor vasculature and therapeutic challenges. Recent Pat. Anticancer Drug Discov..

[127] Kwong J., Kulbe H., Wong D., Chakravarty P., Balkwill F. (2009). An antagonist of the chemokine receptor CXCR4 induces mitotic catastrophe in ovarian cancer cells. Mol. Cancer Ther..

[128] Porvasnik S., Sakamoto N., Kusmartsev S., Eruslanov E., Kim W.J., Cao W., Urbanek C., Wong D., Goodison S., Rosser C.J. (2009). Effects of CXCR4 antagonist CTCE-9908 on prostate tumor growth. Prostate.

[129] Tamamura H., Imai M., Ishihara T., Masuda M., Funakoshi H., Oyake H., Murakami T., Arakaki R., Nakashima H., Otaka A. (1998). Pharmacophore identification of a chemokine receptor (CXCR4) antagonist, T22 ([Tyr5,12, Lys7]-polyphemusin II), which specifically blocks T cell-line-tropic HIV-1 infection. Bioorg. Med. Chem..

[130] Peled A., Abraham M., Avivi I., Rowe J.M., Beider K., Wald H., Tiomkin L., Ribakovsky L., Riback Y., Ramati Y. (2014). The high-affinity CXCR4 antagonist BKT140 is safe and induces a robust mobilization of human CD34+ cells in patients with multiple myeloma. Clin. Cancer Res..

[131] Ling X., Spaeth E., Chen Y., Shi Y., Zhang W., Schober W., Hail N., Konopleva M., Andreeff M. (2013). The CXCR4 antagonist AMD3465 regulates oncogenic signaling and invasiveness *in vitro* and prevents breast cancer growth and metastasis *in vivo*. PLoS ONE.

[132] Singh A.K., Arya R.K., Trivedi A.K., Sanyal S., Baral R., Dormond O., Briscoe D.M., Datta D. (2013). Chemokine receptor trio: CXCR3, CXCR4 and CXCR7 crosstalk via CXCL11 and CXCL12. Cytokine Growth Factor Rev..

[133] Egorova A., Kiselev A., Hakli M., Ruponen M., Baranov V., Urtti A. (2009). Chemokine-derived peptides as carriers for gene delivery to CXCR4 expressing cells. J. Gene Med..

[134] Sebag M. (2012). CCR1 blockade and myeloma bone disease. Blood.

[135] Kitamura T., Fujishita T., Loetscher P., Revesz L., Hashida H., Kizaka-Kondoh S., Aoki M., Taketo M.M. (2010). Inactivation of chemokine (C-C motif) receptor 1 (CCR1) suppresses colon cancer liver metastasis by blocking accumulation of immature myeloid cells in a mouse model. Proc. Natl. Acad. Sci. USA.

[136] Robinson S.C., Scott K.A., Wilson J.L., Thompson R.G., Proudfoot A.E., Balkwill F.R. (2003). A chemokine receptor antagonist inhibits experimental breast tumor growth. Cancer Res..

[137] Yamamoto K., Utsunomiya A., Tobinai K., Tsukasaki K., Uike N., Uozumi K., Yamaguchi K., Yamada Y., Hanada S., Tamura K. (2010). Phase I study of KW-0761, a defucosylated humanized anti-CCR4 antibody, in relapsed patients with adult T-cell leukemia-lymphoma and peripheral T-cell lymphoma. J. Clin. Oncol..

[138] Ogura M., Ishida T., Hatake K., Taniwaki M., Ando K., Tobinai K., Fujimoto K., Yamamoto K., Miyamoto T., Uike N. (2014). Multicenter phase II study of mogamulizumab (KW-0761), a defucosylated anti-CC chemokine receptor 4 antibody, in patients With relapsed peripheral T-cell lymphoma and cutaneous T-cell lymphoma. J. Clin. Oncol..

[139] Rosca E.V., Lal B., Koskimaki J.E., Popel A.S., Laterra J. (2012). Collagen IV and CXC chemokine-derived antiangiogenic peptides suppress glioma xenograft growth. Anticancer Drugs.

[140] Karagiannis E.D., Popel A.S. (2008). Novel anti-angiogenic peptides derived from ELR-containing CXC chemokines. J. Cell. Biochem..

[141] Koskimaki J.E., Karagiannis E.D., Rosca E.V., Vesuna F., Winnard P.T., Raman V., Bhujwalla Z.M., Popel A.S. (2009). Peptides derived from type IV collagen, CXC chemokines, and thrombospondin-1 domain-containing proteins inhibit neovascularization and suppress tumor growth in MDA-MB-231 breast cancer xenografts. Neoplasia.

[142] Vandercappellen J., van Damme J., Struyf S. (2011). The role of the CXC chemokines platelet factor-4 (CXCL4/PF-4) and its variant (CXCL4L1/PF-4var) in inflammation, angiogenesis and cancer. Cytokine Growth Factor Rev..

[143] Wang Z., Huang H. (2013). Platelet factor-4 (CXCL4/PF-4): An angiostatic chemokine for cancer therapy. Cancer Lett..

[144] Kuo J.-H., Chen Y.-P., Liu J.-S., Dubrac A., Quemener C., Prats H., Bikfalvi A., Wu W.-G., Sue S.-C. (2013). Alternative C-terminal helix orientation alters chemokine function: Structure of the anti-angiogenic chemokin, CXCL4L1. J. Biol. Chem..

[145] Dubrac A., Quemener C., Lacazette E., Lopez F., Zanibellato C., Wu W.-G., Bikfalvi A., Prats H. (2010). Functional divergence between 2 chemokines is conferred by single amino acid change. Blood.

[146] Struyf S., Burdick M.D., Proost P., van Damme J., Strieter R.M. (2004). Platelets release CXCL4L1, a nonallelic variant of the chemokine platelet factor-4/CXCL4 and potent inhibitor of angiogenesis. Circ. Res..

[147] Struyf S., Salogni L., Burdick M.D., Vandercappellen J., Gouwy M., Noppen S., Proost P., Opdenakker G., Parmentier M., Gerard C. (2011). Angiostatic and chemotactic activities of the CXC chemokine CXCL4L1 (platelet factor-4 variant) are mediated by CXCR3. Blood.

[148] De Chaisemartin L., Goc J., Damotte D., Validire P., Magdeleinat P., Alifano M., Cremer I., Fridman W.H., Sautes-Fridman C., Dieu-Nosjean M.C. (2011). Characterization of chemokines and adhesion molecules associated with T cell presence in tertiary lymphoid structures in human lung cancer. Cancer Res..

[149] Vandercappellen J., Liekens S., Bronckaers A., Noppen S., Ronsse I., Dillen C., Belleri M., Mitola S., Proost P., Presta M. (2010). The COOH-terminal peptide of platelet factor-4 variant (CXCL4L1/PF-4var47–70) strongly inhibits angiogenesis and suppresses B16 melanoma growth *in vivo*. Mol. Cancer Res..

[150] Singh S., Singh A.P., Sharma B., Owen L.B., Singh R.K. (2010). CXCL8 and its cognate receptors in melanoma progression and metastasis. Future Oncol..

[151] Varney M.L., Singh S., Li A., Mayer-Ezell R., Bond R., Singh R.K. (2011). Small molecule antagonists for CXCR2 and CXCR1 inhibit human colon cancer liver metastases. Cancer Lett..

[152] Agarwal A., Tressel S.L., Kaimal R., Balla M., Lam F.H., Covic L., Kuliopulos A. (2010). Identification of a metalloprotease-chemokine signaling system in the ovarian cancer microenvironment: implications for antiangiogenic therapy. Cancer Res..

[153] Seaton A., Maxwell P.J., Hill A., Gallagher R., Pettigrew J., Wilson R.H., Waugh D.J. (2009). Inhibition of constitutive and cxc-chemokine-induced NF-κB activity potentiates ansamycin-based HSP90-inhibitor cytotoxicity in castrate-resistant prostate cancer cells. Br. J. Cancer.

[154] Wang P., Yang X., Xu W., Li K., Chu Y., Xiong S. (2010). Integrating individual functional moieties of CXCL10 and CXCL11 into a novel chimeric chemokine leads to synergistic antitumor effects: A strategy for chemokine-based multi-target-directed cancer therapy. Cancer Immunol. Immunother..

